# Supplementary material for: Modeling glioblastoma heterogeneity as a dynamic network of cell states
Source: Mol Syst Biol. 2021 Sep 16;17(9):e10105. doi: 10.15252/msb.202010105 (PMC8444284; doi:10.15252/msb.202010105)
Supplement: Supplementary file 6 — Source Data for Figure 5 [file MSB-17-e10105-s004.zip › Figure5A_sourcedata/GSEA_3017/nosigdb_stateF.GseaPreranked.1622795015644/RICHARDS_ZHONG_NATURE_2018_MICROGLIA_UPREG.html]

Details for gene set RICHARDS\_ZHONG\_NATURE\_2018\_MICROGLIA\_UPREG[GSEA]

|  || Dataset | state33017 |
| Phenotype | NoPhenotypeAvailable |
| Upregulated in class | na\_pos |
| GeneSet | RICHARDS\_ZHONG\_NATURE\_2018\_MICROGLIA\_UPREG |
| Enrichment Score (ES) | 0.4977337 |
| Normalized Enrichment Score (NES) | 2.9699788 |
| Nominal p-value | 0.0 |
| FDR q-value | 0.0 |
| FWER p-Value | 0.0 |
Table: GSEA Results Summary

  

Fig 1: Enrichment plot: RICHARDS\_ZHONG\_NATURE\_2018\_MICROGLIA\_UPREG      
 Profile of the Running ES Score & Positions of GeneSet Members on the Rank Ordered List

  

| PROBE | GENE SYMBOL | GENE\_TITLE | RANK IN GENE LIST | RANK METRIC SCORE | RUNNING ES | CORE ENRICHMENT || 1 | NEAT1 |  |  | 0 | 2.864 | 0.0594 | Yes |
| 2 | APOE |  |  | 17 | 1.244 | 0.0778 | Yes |
| 3 | FTL |  |  | 32 | 1.036 | 0.0929 | Yes |
| 4 | ITPR2 |  |  | 34 | 1.024 | 0.1136 | Yes |
| 5 | PLTP |  |  | 35 | 1.020 | 0.1348 | Yes |
| 6 | A2M |  |  | 42 | 0.974 | 0.1522 | Yes |
| 7 | SAT1 |  |  | 45 | 0.957 | 0.1711 | Yes |
| 8 | BST2 |  |  | 55 | 0.910 | 0.1859 | Yes |
| 9 | SESN3 |  |  | 56 | 0.908 | 0.2047 | Yes |
| 10 | SPP1 |  |  | 57 | 0.898 | 0.2233 | Yes |
| 11 | GLUL |  |  | 69 | 0.837 | 0.2356 | Yes |
| 12 | SERPINB6 |  |  | 83 | 0.791 | 0.2460 | Yes |
| 13 | IFITM2 |  |  | 103 | 0.749 | 0.2528 | Yes |
| 14 | B2M |  |  | 110 | 0.736 | 0.2653 | Yes |
| 15 | NPC2 |  |  | 114 | 0.728 | 0.2790 | Yes |
| 16 | GNG7 |  |  | 115 | 0.726 | 0.2940 | Yes |
| 17 | BRI3 |  |  | 121 | 0.716 | 0.3066 | Yes |
| 18 | BTG2 |  |  | 124 | 0.708 | 0.3203 | Yes |
| 19 | ANXA4 |  |  | 127 | 0.703 | 0.3340 | Yes |
| 20 | FCGR2A |  |  | 134 | 0.688 | 0.3455 | Yes |
| 21 | MAF |  |  | 136 | 0.688 | 0.3593 | Yes |
| 22 | ITM2C |  |  | 160 | 0.649 | 0.3621 | Yes |
| 23 | ITM2B |  |  | 181 | 0.610 | 0.3656 | Yes |
| 24 | NINJ1 |  |  | 182 | 0.610 | 0.3782 | Yes |
| 25 | TIMP2 |  |  | 196 | 0.590 | 0.3844 | Yes |
| 26 | HERPUD1 |  |  | 197 | 0.590 | 0.3967 | Yes |
| 27 | HTRA1 |  |  | 229 | 0.568 | 0.3942 | Yes |
| 28 | CEBPD |  |  | 234 | 0.566 | 0.4040 | Yes |
| 29 | BLVRB |  |  | 235 | 0.565 | 0.4158 | Yes |
| 30 | TPP1 |  |  | 286 | 0.521 | 0.4035 | Yes |
| 31 | ASAH1 |  |  | 293 | 0.515 | 0.4114 | Yes |
| 32 | ATP6V0E1 |  |  | 303 | 0.510 | 0.4178 | Yes |
| 33 | LAMP2 |  |  | 308 | 0.506 | 0.4265 | Yes |
| 34 | GSTK1 |  |  | 312 | 0.503 | 0.4355 | Yes |
| 35 | IL6ST |  |  | 321 | 0.500 | 0.4422 | Yes |
| 36 | RNF13 |  |  | 330 | 0.496 | 0.4488 | Yes |
| 37 | PLD3 |  |  | 337 | 0.494 | 0.4563 | Yes |
| 38 | DPP7 |  |  | 342 | 0.490 | 0.4646 | Yes |
| 39 | LIPA |  |  | 364 | 0.483 | 0.4649 | Yes |
| 40 | GATM |  |  | 375 | 0.480 | 0.4703 | Yes |
| 41 | CTSL |  |  | 376 | 0.479 | 0.4802 | Yes |
| 42 | SORL1 |  |  | 402 | 0.469 | 0.4784 | Yes |
| 43 | METTL7A |  |  | 403 | 0.469 | 0.4881 | Yes |
| 44 | PSAP |  |  | 418 | 0.463 | 0.4912 | Yes |
| 45 | LAMP1 |  |  | 440 | 0.455 | 0.4910 | Yes |
| 46 | GAS6 |  |  | 463 | 0.445 | 0.4901 | Yes |
| 47 | GRN |  |  | 474 | 0.443 | 0.4946 | Yes |
| 48 | LPIN2 |  |  | 488 | 0.438 | 0.4977 | Yes |
| 49 | RAB31 |  |  | 580 | 0.404 | 0.4641 | No |
| 50 | FOS |  |  | 592 | 0.401 | 0.4674 | No |
| 51 | DOCK4 |  |  | 668 | 0.380 | 0.4406 | No |
| 52 | TNFRSF1A |  |  | 686 | 0.374 | 0.4405 | No |
| 53 | S100A11 |  |  | 692 | 0.373 | 0.4460 | No |
| 54 | RAB29 |  |  | 706 | 0.369 | 0.4476 | No |
| 55 | ADAM9 |  |  | 722 | 0.365 | 0.4483 | No |
| 56 | GAL3ST4 |  |  | 738 | 0.362 | 0.4489 | No |
| 57 | RBMS1 |  |  | 761 | 0.355 | 0.4461 | No |
| 58 | SIPA1L2 |  |  | 766 | 0.354 | 0.4516 | No |
| 59 | TPT1 |  |  | 786 | 0.350 | 0.4501 | No |
| 60 | KCTD12 |  |  | 808 | 0.345 | 0.4475 | No |
| 61 | FCGRT |  |  | 814 | 0.344 | 0.4524 | No |
| 62 | ZFP36L2 |  |  | 850 | 0.336 | 0.4432 | No |
| 63 | CD81 |  |  | 947 | 0.318 | 0.4055 | No |
| 64 | CPVL |  |  | 1019 | 0.307 | 0.3791 | No |
| 65 | INPP5D |  |  | 1069 | 0.297 | 0.3626 | No |
| 66 | RNF213 |  |  | 1091 | 0.294 | 0.3590 | No |
| 67 | LGMN |  |  | 1110 | 0.291 | 0.3567 | No |
| 68 | MFSD1 |  |  | 1144 | 0.286 | 0.3474 | No |
| 69 | PTPRE |  |  | 1243 | 0.275 | 0.3079 | No |
| 70 | CTSD |  |  | 1245 | 0.275 | 0.3131 | No |
| 71 | SGK1 |  |  | 1283 | 0.270 | 0.3017 | No |
| 72 | IFNGR1 |  |  | 1289 | 0.269 | 0.3050 | No |
| 73 | NEU1 |  |  | 1296 | 0.269 | 0.3078 | No |
| 74 | CTSA |  |  | 1385 | 0.258 | 0.2725 | No |
| 75 | POLD4 |  |  | 1430 | 0.255 | 0.2575 | No |
| 76 | MGAT4A |  |  | 1448 | 0.252 | 0.2549 | No |
| 77 | JUNB |  |  | 1466 | 0.251 | 0.2522 | No |
| 78 | APLP2 |  |  | 1468 | 0.250 | 0.2570 | No |
| 79 | PFN1 |  |  | 1582 | -0.282 | 0.2107 | No |
| 80 | ZFP36L1 |  |  | 1590 | -0.284 | 0.2133 | No |
| 81 | TACC1 |  |  | 1701 | -0.319 | 0.1692 | No |
| 82 | GPN3 |  |  | 1739 | -0.335 | 0.1591 | No |
| 83 | LIMS1 |  |  | 1807 | -0.365 | 0.1357 | No |
| 84 | C12orf75 |  |  | 1832 | -0.380 | 0.1325 | No |
| 85 | IER2 |  |  | 2034 | -0.524 | 0.0506 | No |
| 86 | MIS18BP1 |  |  | 2183 | -0.903 | 0.0010 | No |
| 87 | HIST1H1C |  |  | 2243 | -1.485 | 0.0046 | No |
Table: GSEA details [plain text format]

  

Fig 2: RICHARDS\_ZHONG\_NATURE\_2018\_MICROGLIA\_UPREG: Random ES distribution      
 Gene set null distribution of ES for **RICHARDS\_ZHONG\_NATURE\_2018\_MICROGLIA\_UPREG**
